# Global knockdown of glutamate decarboxylase 67 elicits emotional abnormality in mice

**DOI:** 10.1186/s13041-020-00713-2

**Published:** 2021-01-07

**Authors:** Shigeo Miyata, Toshikazu Kakizaki, Kazuyuki Fujihara, Hideru Obinata, Touko Hirano, Junichi Nakai, Mika Tanaka, Shigeyoshi Itohara, Masahiko Watanabe, Kenji F. Tanaka, Manabu Abe, Kenji Sakimura, Yuchio Yanagawa

**Affiliations:** 1 Department of Genetic and Behavioral Neuroscience, Gunma University Graduate School of Medicine, Maebashi, 371-8511 Japan; 2 Laboratory for Analytical Instruments, Education and Research Support Center, Gunma University Graduate School of Medicine, Maebashi, 371-8511 Japan; 3 Division of Oral Physiology, Department of Oral Function and Morphology, Tohoku University Graduate School of Dentistry, Sendai, 980-8575 Japan; 4 Laboratory for Behavioral Genetics, RIKEN Brain Science Institute, Wako, 351-0198 Japan; 5 Department of Anatomy, Faculty of Medicine, Hokkaido University, Sapporo, 060‐8638 Japan; 6 Department of Neuropsychiatry, Keio University School of Medicine, Tokyo, 160-8582 Japan; 7 Department of Animal Model Development, Brain Research Institute, Niigata University, Niigata, 951-8585 Japan

**Keywords:** Animal model, Auditory function, Behavior, GABA, Glutamate decarboxylase, Knockdown mice, Tetracycline-controlled gene expression

## Abstract

Reduced expression of glutamate decarboxylase 67 (GAD67), encoded by the *Gad1* gene, is a consistent finding in postmortem brains of patients with several psychiatric disorders, including schizophrenia, bipolar disorder and major depressive disorder. The dysfunction of GAD67 in the brain is implicated in the pathophysiology of these psychiatric disorders; however, the neurobiological consequences of GAD67 dysfunction in mature brains are not fully understood because the homozygous *Gad1* knockout is lethal in newborn mice. We hypothesized that the tetracycline-controlled gene expression/suppression system could be applied to develop global GAD67 knockdown mice that would survive into adulthood. In addition, GAD67 knockdown mice would provide new insights into the neurobiological impact of GAD67 dysfunction. Here, we developed *Gad1*^tTA/STOP−tetO^ biallelic knock-in mice using *Gad1*^STOP−tetO^ and *Gad1*^tTA^ knock-in mice, and compared them with *Gad1*^+/+^ mice. The expression level of GAD67 protein in brains of *Gad1*^tTA/STOP−tetO^ mice treated with doxycycline (Dox) was decreased by approximately 90%. The GABA content was also decreased in the brains of Dox-treated *Gad1*^tTA/STOP−tetO^ mice. In the open-field test, Dox-treated *Gad1*^tTA/STOP−tetO^ mice exhibited hyper-locomotor activity and decreased duration spent in the center region. In addition, acoustic startle responses were impaired in Dox-treated *Gad1*^tTA/STOP−tetO^ mice. These results suggest that global reduction in GAD67 elicits emotional abnormalities in mice. These GAD67 knockdown mice will be useful for elucidating the neurobiological mechanisms of emotional abnormalities, such as anxiety symptoms associated with psychiatric disorders.

## Introduction

γ-Aminobutyric acid (GABA), a major inhibitory neurotransmitter, regulates a variety of biological functions. GABA is synthesized from glutamate by glutamate decarboxylase (GAD) existing two isoforms with different molecular weights, 67 kDa (GAD67) and 65 kDa (GAD65), which are independently encoded by the *Gad1* and *Gad2* genes, respectively [1, 2]. Since GAD67 and GAD65 proteins have different subcellular distributions and a cofactor association [3–6], the physiological roles of GAD67 and GAD65 may be different in the brain.

Decreased expression of the full-length *GAD1* transcript and GAD67 protein is one of the most consistent findings in the brains of subjects with several psychiatric disorders, including schizophrenia, bipolar disorder and major depressive disorder [7–12]. Alternative splicing of *GAD1* and the epigenetic state may play roles in brain development and the risk of schizophrenia [12]. Recent studies with whole exome sequencing of schizophrenic patients identified missense mutation mapping at the *GAD1* gene, which caused a reduction in GAD67 enzymatic activity by ~ 30% due to impaired homodimerization [13, 14]. Therefore, the functional impairment of GAD67 associated with genetic mutations is involved in the neurobiological mechanisms of several psychiatric disorders. To elucidate the physiological roles of GAD67 in mature brains, a study using transgenic animals with reduced GAD67 expression would be helpful. Because homozygous *Gad1* knockout (*Gad1*^−/−^) is lethal in newborn mice [15], conditional *Gad1*^−/−^ mice generated with a Cre-loxP strategy have often been used for neurobiological studies [16–20]. These studies have provided much information about the function of GAD67 in targeted cells. However, behavioral and neurochemical consequences of the global dysfunction of GAD67 in mature brains are not fully understood. Several studies have been performed to investigate GAD67 function using mice with GAD67 haplodeficiency; however, physiological changes, such as GABA reduction in the brain and behavioral abnormalities, were mild in those mice [15, 21–24].

The tetracycline-controlled gene expression/suppression system allows reduction in a gene of interest by administration of antibiotic tetracyclines and its derivative doxycycline (Dox) [25]. This system requires two distinct transgenic mice: one has a cell type-specific promoter driving a tetracycline-controlled transcriptional activator (tTA)-expressing allele, and the other has a tetracycline operator site (tetO) binding to tTA and driving the expression of the gene of interest. By crossing them, a biallelic knock-in mouse (tTA/tetO) can be obtained with a tTA-mediated gene induction system, which can be turned off by the administration of Dox.

**Fig. 1:**
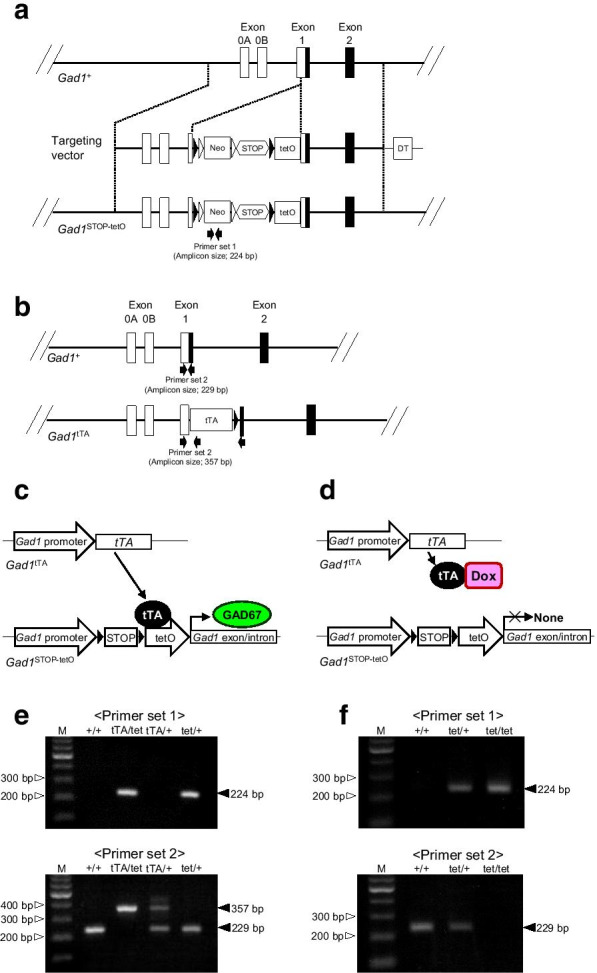
Doxycycline (Dox)-regulated *Gad1* suppression system. **a** Generation of *Gad1*^STOP−tetO^ knock-in mice. Schematic diagram depicting *Gad1* genomic DNA (*Gad1*^+^), targeting vector, and *Gad1* genomic DNA inserted into the Neo-STOP-tetO cassette (*Gad1*^STOP−tetO^). Arrows indicate the PCR primers (Primer set 1). **b** Schematic representation of *Gad1* genomic DNA (*Gad1*^+^) and DNA containing the tetracycline-controlled transactivator (tTA) gene (*Gad1*^tTA^). Arrows indicate the PCR primers (Primer set 2). **c**, **d** Schematic diagram of the Dox-regulated *Gad1* suppression system in *Gad1*^tTA/STOP−tetO^ mice. Before Dox treatment, tTA binds to the tetracycline operator site (tetO) and promotes *Gad1* transcription and GAD67 production (**c**). Dox treatment interferes with tTA binding to tetO and suppresses *Gad1* transcription (**d**). **e**, **f** PCR genotyping. Representative results of PCR genotyping for *Gad1*^tTA/STOP−tetO^ mice (**e**), *Gad1*^STOP−tetO/STOP−tetO^ mice (**f**) and their littermates. M; 100-bp size marker, +/+; *Gad1*^+/+^, tTA/tet; *Gad1*^tTA/STOP−tetO^, tTA/ + ; *Gad1*^tTA/+^, tet/ + ; *Gad1*^STOP−tetO/+^, tet/tet; *Gad1*^STOP−tetO/STOP−tetO^

**Fig. 2:**
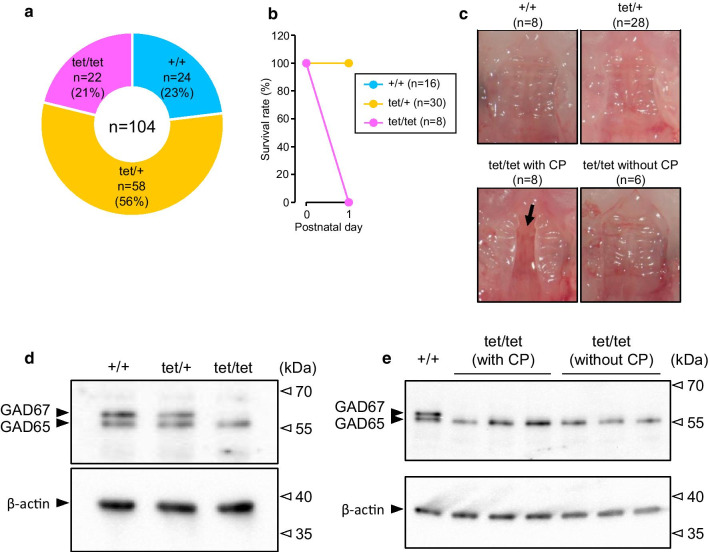
Characterization of homozygous *Gad1*^STOP−tetO/STOP−tetO^ mice. **a** The number of mouse pups born from heterozygous *Gad1*^STOP−tetO/+^ parents. +/+; *Gad1*^+/+^, tet/+; *Gad1*^STOP−tetO/+^, tet/tet; *Gad1*^STOP−tetO/STOP−tetO^. **b** The survival rates of mouse pups born from heterozygous *Gad1*^STOP−tetO/+^ parents. **c** Microscopic images of the palate of mice. The arrow indicates a cleft palate (CP). **d** Representative western blot analysis of the expression of GAD65/67 and β-actin proteins in the brains of *Gad1*^+/+^, *Gad1*^STOP−tetO/+^ and *Gad1*^STOP−tetO/STOP−tetO^ mice. **e** Western blot analysis of the expression of GAD65/67 and β-actin proteins in the brains of 3 *Gad1*^STOP−tetO/STOP−tetO^ mice with and without CP. The *Gad1*^+/+^ mouse protein sample was used as a positive control for GAD67 immunoblotting

We hypothesized that the tetracycline-controlled gene expression/suppression system could be applied to generate transgenic mice able to postnatally knockdown GAD67. In brief, two knock-in mice were generated: (1) *Gad1*^STOP−tetO^ knock-in mice, which were generated by inserting the tetO sequence following the STOP sequence upstream of the *Gad1* translation initiation site (Fig. 1a), and (2) *Gad1*^tTA^ knock-in mice [26], which express tTA proteins under the control of an endogenous *Gad1* promoter (Fig. 1b). The *Gad1*^tTA/STOP−tetO^ mice can produce GAD67 protein (Fig. 1c) and survive into adulthood. In addition, the expression of GAD67 protein in the *Gad1*^tTA/STOP−tetO^ mice is suppressed by treatment with Dox (Fig. 1d). Therefore, Dox-treated *Gad1*^tTA/STOP−tetO^ mice can be used as GAD67 knockdown mice for studying the behavioral and neurochemical consequences of the global loss of GAD67 in mature brains.

The major aim of this study was to examine the phenotypes of mice with GAD67 suppression at adult stage. In this study, we successfully developed *Gad1*^STOP−tetO^ knock-in mice and the subsequent *Gad1*^tTA/STOP−tetO^ mice. Herein, we report the behavioral abnormalities elicited by the global knockdown of GAD67 in mice.

## Methods

### Ethics

This study was performed in accordance with the Guidelines for Animal Experimentation at Gunma University Graduate School of Medicine and was approved by the Gunma University Ethics Committee (Permit number: 14-006 and 19-009). Every effort was made to minimize the number of animals used and their suffering.

### Experimental design

We first generated *Gad1*^STOP−tetO^ knock-in mice and assessed the *Gad1* knockout phenotypes (GAD67 deletion, neonatal death and cleft palate) of homozygous *Gad1*^STOP−tetO/STOP−tetO^ mice to confirm the elimination of *Gad1* gene function by inserting the Neo-STOP-tetO cassette. We then crossed heterozygous *Gad1*^tTA/+^ and *Gad1*^STOP−tetO/+^ mice to obtain *Gad1*^tTA/STOP−tetO^ mice and confirmed tTA-mediated GAD67 expression. Afterward, we evaluated whether tTA-mediated GAD67 expression was suppressed by treatment with Dox. It has been reported that GAD67 haplodeficient mice demonstrate an approximately 40% reduction in GAD67 protein in the brain compared with wild-type mice [27]. Therefore, we judged that GAD67 knockdown mice were successfully developed when the expression level of GAD67 protein in the brain was reduced by more than 40%. These experiments were performed in mice of both sexes.

In the behavioral tests, male mice were only used. We prepared two independent cohorts comparing *Gad1*^+/+^ mice and *Gad1*^tTA/STOP−tetO^ mice at the ages of 8 to 10 weeks. One cohort was used for assessing the body weights, motor coordination performance, and GABA and glutamate contents in their brains. The body weights and motor coordination performance of the mice were measured 3 weeks after starting Dox treatment. Immediately after the motor coordination test, the mice were killed by decapitation, and the frontal cortex (FCX), hippocampus (HIP) and cerebellum (CER) were quickly dissected. The collected tissues were immediately frozen in liquid nitrogen and stored at − 80 °C until use. The frozen tissues were used for measuring GABA and glutamate contents. Another cohort was used for the open-field test and PPI test. Three weeks after treatment with Dox, the open-field test was conducted. After testing, the mice were returned to their home cage, and treatment with Dox was continued. Two days after the open-field test, the acoustic startle responses and prepulse inhibition (PPI) responses were assessed in the mice.

### Animals

To generate *Gad1*^STOP−tetO^ knock-in mice, we constructed the *Gad1* targeting vector by linking the following elements in tandem: the 4.7-kb 5′-homology arm, 3.4-kb Neo-STOP-tetO cassette [28], 5.9-kb 3′-homology arm, and the MC1 promoter-driven diphtheria toxin A subunit gene (DT) (Fig. 1a). The Neo-STOP-tetO cassette comprised the 1.7-kb PGK-Neo cassette, a 1.3-kb STOP sequence, and a 0.5-kb tetO site. The targeting vector was designed to insert the Neo-STOP-tetO cassette just upstream of the *Gad1* translation initiation site. We used B6-derived embryonic cells for homologous recombination. From 179 G418-resistant clones, we obtained 46 recombinant clones. Germline-transmitted offspring were established as *Gad1*^STOP−tetO^ knock-in mice (Fig. 1a).

The generation of *Gad1*^tTA^ knock-in mice has already been described [26]. In these mice, the *tTA2* cDNA followed by the SV40 polyadenylation signal was inserted into exon 1 of the *Gad1* gene in frame with the translation initiation codon, and the tTA protein was expressed under the control of an endogenous *Gad1* promoter (Fig. 1b).

Heterozygous mice carrying one STOP-tetO allele (*Gad1*^STOP−tetO/+^ mice) were crossed with heterozygous mice carrying one tTA allele (*Gad1*^tTA/+^ mice) to obtain four genotypes: *Gad1*^+/+^, *Gad1*^tTA/+^, *Gad1*^STOP−tetO/+^ and *Gad1*^tTA/STOP−tetO^ mice. To prevent the expression of GAD67 protein, we administered 100 mg of Dox per kg of regular mouse chow CE-2 (CLEA Japan, Inc.).

The animals were housed at 2–3 mice per cage (16.5 × 27 × 12.5 (H) cm) and had free access to food and water. The animal rooms for breeding and experiments were maintained at 22 ± 3 °C with a 12-h light–dark cycle (lights on at 6:00, lights off at 18:00).

### Genotyping

Genotyping of the transgenic mice was performed by PCR using tail genomic DNA with SapphireAmp Fast PCR master mix (Takara Bio Inc., Japan) and the specific primer sets. Primer set 1 determined the existence of the *Neo* allele (Fig. 1a); the sequences were Neo-F, 5′-CAGCTGTGCTCGACGTTGTC-3′ and Neo-R, 5′-AAGACCGGCTTCCATCCGAG-3′. Primer set 2 determined the existence of the *Gad1* and *tTA*-inserted *Gad1* alleles (Fig. 1b); the sequences were Gad1-F, 5′-TGGTCTCCCTTCTGTCTCCGA-3′, Gad1-R, 5′-TGTAGGGCGCAGGTTGGTAG-3′, and tTA-R, 5′-GGGCAAAAGTGAGTATGGTGCC-3′. After amplification, 5 μL of each reaction mixture and a size marker (Loading Quick 100 bp DNA Ladder, TOYOBO Co. Ltd, Osaka, Japan) were analyzed by 2% agarose gel electrophoresis, and the bands were then visualized by ethidium bromide staining. The lengths of the amplified DNA fragments were 224 bp (*Neo* allele), 229 bp (*Gad1* allele) and 357 bp (*tTA*-inserted *Gad1* allele) (Fig. 1e, f).

### Palate formation

Mouse neonates were killed by decapitation, and the lower jaw was removed. The cleft palate of the mouse was determined under a stereoscopic microscope.

### Immunoblot analysis

The mice were killed by decapitation. The brain hemispheres of neonates and the FCX, HIP and CER of adult mice were quickly dissected on an ice-cold stainless plate. The tissues were immediately frozen in liquid nitrogen and stored at − 80 °C until use. The frozen tissues were homogenized in ice-cold buffered sucrose (0.32 M) solution containing 20 mM Tris–HCl (pH 7.5) and protease inhibitor cocktail (P8340, Sigma-Aldrich, Inc.). The homogenates were centrifuged at 1000×*g* for 10 min at 4 °C, and the supernatants were collected as the protein samples. The protein concentrations were determined using a TaKaRa BCA Protein Assay Kit (T9300A, Takara Bio Inc., Japan).

The protein samples were diluted with electrophoresis sample buffer. Proteins (1.5 μg) were separated by 8% SDS-polyacrylamide gels and transferred to a PVDF membrane. Blots were probed with respective antibodies to GAD65/67 (1:1000, rabbit polyclonal antibody) [29] and GAD67 (1:1000, mouse monoclonal antibody, Millipore, Code No. MAB5406). Immunoblots were developed using horseradish peroxidase-conjugated secondary antibodies (GE Healthcare) and then detected with chemiluminescence reagents (ECL prime, GE Healthcare) and visualized by the Light Capture AE-9672 (ATTO Co., Ltd.). After the detection of immunoblots, the blotting membranes were washed with PBS several times and reprobed with a mouse monoclonal antibody to β-actin (1:10,000, Medical & Biological Laboratories Co. Ltd., Code No. M177-3). The immunoblots of β-actin were developed and visualized by the same protocol described above. The density of the bands was determined using ImageJ software. The band densities of β-actin were used as the loading control. The relative expression level of GAD67 to β-actin was calculated and used for comparisons between the genotypes.

### Double-label immunofluorescence analysis

Deeply anesthetized mice by continuous inhalation of isoflurane were fixed by perfusion with Mildform 10 N (containing 3.7–4.3 w/w% formaldehyde; FUJIFILM Wako Pure Chemical Co., Osaka, Japan) through the left ventricle. The brain was removed and postfixed in Mildform 10 N overnight at 4 °C. The brain hemispheres were cut into 50-μm-thick sagittal sections by a vibrating blade tissue slicer (Neo-LinearSlicer MT, Dosaka EM Co., Ltd., Kyoto, Japan).

Free-floating immunostaining was performed by using a VECTOR M.O.M.® (Mouse on Mouse) Immunodetection Kit (BMK-2202, Vector Laboratories Inc., USA). The sections were incubated overnight at room temperature in the 1st primary antibodies against GAD67 (1:300, mouse monoclonal antibody, MAB5406, Millipore) and parvalbumin (PV) (1:300, guinea pig polyclonal antibody, PV-GP-Af1000, Frontier Institute Co. Ltd., Hokkaido, Japan) with the M.O.M. Blocking reagent after preincubation with 0.3% Triton X-100 in PBS. After rinsing, the sections were incubated in the M.O.M. Biothinylated Anti-Mouse IgG Reagent (1:300) with a secondary antibody (1:300, goat anti-guinea pig IgG conjugated with AlexaFluor488, A-11073, Invitrogen) for 30 min at room temperature. After rinsing, the sections were incubated in a solution containing Streptavidin-DyLight649 (1:50, SA-5649, Vector Lab.) and DAPI (1:500, D523, Dojindo Laboratories, Japan) for 30 min at room temperature. The stained sections were mounted on MAS-coated glass slides (Matsunami Glass Ind., Ltd., Osaka, Japan) with Fluoromount (K024, Diagnostic BioSystems, USA). Fluorescence images were captured with a fluorescence digital microscope (BZ-X810, Keyence, Osaka, Japan).

Three independent mice in the respective groups were assessed. The names of brain regions were referenced to the Allen Mouse Brain Atlas (https://alleninstitute.org/).

### Motor coordination test

The performance of motor coordination in mice was tested by a rotarod apparatus (Ugo Basile, Comerio, Italy) according to a previous report [16]. Briefly, each mouse was placed in a separate lane of the apparatus on a rotating cylinder (3 cm diameter) at 20 rounds per minute. The latency until the mouse fell from the cylinder (up to 120 s) was recorded in three consecutive trials with 2–3 min intervals, and the median latency was used for the following analysis. If the mouse did not fall within 120 s, the latency to fall was recorded as 120 s.

### Open-field test

Each mouse was placed in the center of an open-field apparatus (50 cm × 50 cm × 40 (H) cm) that was illuminated by light-emitting diodes (30 lx at the center of the field) and allowed to move freely for 5 min. The data were collected and analyzed using ImageJ OF4 (O’Hara & Co., Ltd., Tokyo, Japan), which is modified software that is also based on the public domain ImageJ program. The procedure was performed according to our previous report [30].

### Acoustic startle response and PPI test

An acoustic startle reflex measurement system (O’Hara & Co., Ltd., Tokyo, Japan) was used. The startle response was assessed with various stimulus intensities. Five times of 70 to 120 dB (70, 75, 80, 85, 90, 95, 100, 110, and 120 dB) white noise stimuli (40 ms) were presented in quasi-random order and random intertrial intervals (10–20 s). In the PPI session, mice experienced five trial types: no stimulus; startle stimulus (120 dB, 40 ms) only; prepulse 70 dB (20 ms, lead time 100 ms) and pulse 120 dB; prepulse 75 dB (20 ms, lead time 100 ms) and pulse 120 dB; and prepulse 80 dB (20 ms, lead time 100 ms) and pulse 120 dB. Each trial was repeated 10 times in quasi-random order and random intertrial intervals (10–20 s). PPI was defined as the percent decline of the startle response: 100 − [(startle amplitude after prepulse and pulse)/(startle amplitude after pulse only)] × 100. The procedure was performed according to our previous report [19].

### GABA and glutamate contents in the brains

The frozen tissues were weighed and then homogenized by BioMasher II (Nippi, Inc., Tokyo, Japan) in 500 μL of 0.1% formic acid in acetonitrile (Wako, Tokyo, Japan) containing an internal standard 2-morpholinoethanesulfonic acid (2-MES; Dojindo, Tokyo, Japan). The standard was spiked at a final concentration of 10 μM. The homogenates were centrifuged at 15,000×*g* for 15 min at 4 °C, and then, the supernatants were collected and filtered through an ISOLUTE PLD + column (Biotage Japan Ltd., Tokyo, Japan). The 40 μL filtrates were lyophilized and stored at − 20 °C.

At the time of analysis, the lyophilized samples were dissolved in 1.25 mL of ultrapure water. The prepared sample solutions (3 μL) were then injected on ultra-performance liquid chromatograph coupled to triple-quadrupole mass spectrometer (LC/MS) (LCMS-8050; Shimadzu, Kyoto, Japan). The chromatographic conditions were according to the Shimadzu method package using a pentafluorophenylpropyl (PFPP) column. The MS settings, data acquisition and data analysis were in accordance with the manufacturer’s instructions for analyzing Primary Metabolites version 2.0 (Cat. #: 225-24865A, Shimadzu). The relative values of metabolites from the internal standard 2-MES and the weight of corresponding tissues were calculated and used for the following data analysis.

Standard solutions containing GABA (A2129, Sigma-Aldrich Co. LLC., USA) and l-glutamic acid (G1251, Sigma-Aldrich) at dose ranges of 0.01–3 μmol/L and 0.03–10 μmol/L, respectively, with internal standard 2-MES (10 μM) were also applied to the LC/MS system. The concentrations of GABA and glutamate in the sample solutions were determined by the peak heights of the chromatogram. The GABA and glutamate contents per the corresponding tissue weights were calculated.

### Statistical analysis

Statistical analyses were conducted using BellCurve for Excel ver. 3.20 (Social Survey Research Information Co., Ltd., Tokyo, Japan). Significant differences among the multiple groups were analyzed by the Bonferroni multiple comparison test after one-way analysis of variance (ANOVA). Significant differences between two groups were analyzed by Student’s *t*-test. The factorial comparisons in some experiments were performed by two-way ANOVA. Data are expressed as the mean with standard error (SE). Statistical significance was defined as a *p* value less than 0.05.

## Results

### Generation of *Gad1*^STOP−tetO^ knock-in mice

To confirm the elimination of function of the *Gad1* gene by inserting the Neo-STOP-tetO cassette, we first generated homozygous *Gad1*^STOP−tetO/STOP−tetO^ mice by crossing heterozygous *Gad1*^STOP−tetO/+^ parents and assessed whether the *Gad1*^STOP−tetO/STOP−tetO^ mouse showed the *Gad1* knockout phenotypes. *Gad1*^STOP−tetO/STOP−tetO^ mice, *Gad1*^STOP−tetO/+^ mice and *Gad1*^+/+^ mice were born at the expected Mendelian frequency (Fig. 2a). Then, mouse pups were divided into two groups to determine the survival rates and palate formation. All *Gad1*^STOP−tetO/STOP−tetO^ mice died within 1 day after birth, but *Gad1*^STOP−tetO/+^ mice and *Gad1*^+/+^ mice survived (Fig. 2b). All *Gad1*^STOP−tetO/+^ and *Gad1*^+/+^ mice formed normal palate. However, 57% of *Gad1*^STOP−tetO/STOP−tetO^ mice exhibited a cleft palate (Fig. 2c). These phenotypes are the same as those of *Gad1*^−/−^ mice previously reported [31]. Western blot analyses demonstrated that the expression of GAD67 protein in the brain was abolished in *Gad1*^STOP−tetO/STOP−tetO^ mice with or without cleft palate (Fig. 2d, e). These observations indicate that the insertion of the Neo-STOP-tetO cassette following the *Gad1* promoter eliminates the function of the *Gad1* gene in mice.

### Development of GAD67 knockdown mice

**Fig. 3:**
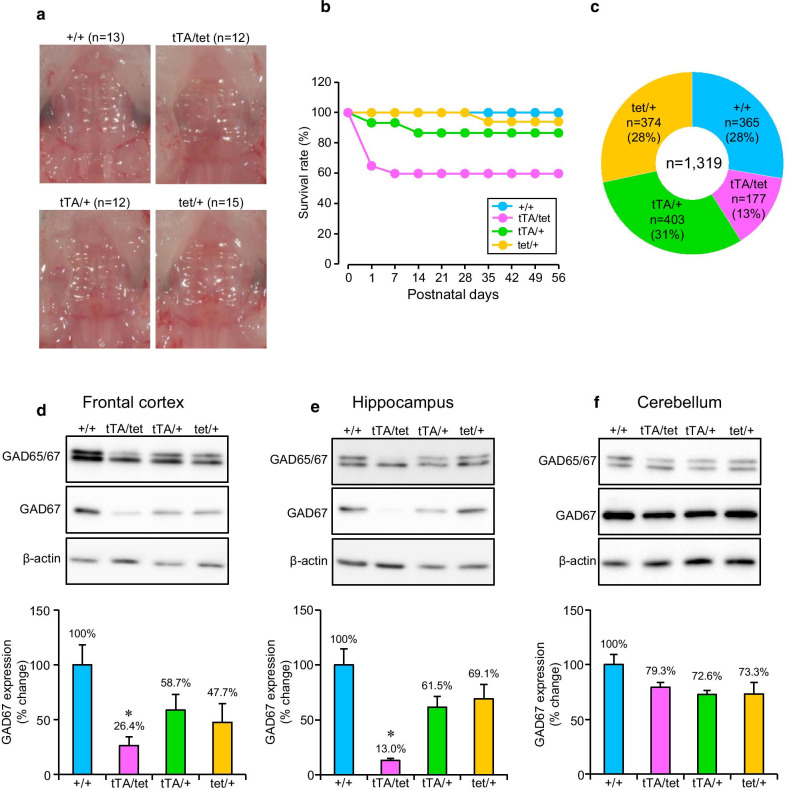
Characterization of *Gad1*^tTA/STOP−tetO^ mice in the absence of Dox treatment. **a** Microscopic images of the palates of mice. **b** The survival rates of *Gad1*^+/+^ (+/+), *Gad1*^tTA/STOP−tetO^ (tTA/tet), *Gad1*^tTA/+^ (tTA/+) and *Gad1*^STOP−tetO/+^ (tet/+) mice. The number of mice at postnatal day 0 was 15 (+/+), 20 (tTA/tet), 15 (tTA/+) and 17 (tet/+). **c** The number of mice with four genotypes at postnatal days 21–28. **d**–**f** Representative western blot analysis of the expression of GAD65/67 and β-actin proteins in the frontal cortex (**d**), the hippocampus (**e**) and the cerebellum (**f**) of *Gad1*^+/+^, *Gad1*^tTA/STOP−tetO^, *Gad1*^tTA/+^ and *Gad1*^STOP−tetO/+^ mice. β-Actin was used as a loading control. Three independent mice of the respective genotypes were examined. The band densities of GAD67 and β-actin were quantified by ImageJ software. GAD67 protein levels were normalized to β-actin protein expression, and the % change was calculated relative to *Gad1*^+/+^ mice. The means with SE are demonstrated as columns. *p < 0.05 vs. the value of *Gad1*^+/+^ mice (Bonferroni test)

We then crossed heterozygous male *Gad1*^tTA/+^ mice and female *Gad1*^STOP−tetO/+^ mice to generate *Gad1*^tTA/STOP−tetO^ biallelic knock-in mice. Mice with four genotypes (*Gad1*^+/+^, *Gad1*^tTA/+^, *Gad1*^STOP−tetO/+^, *Gad1*^tTA/STOP−tetO^) were born at the expected Mendelian frequency (Fig. 3*legend*). Palate formation in mouse neonates with four genotypes was observed, but none of them exhibited the cleft palate (Fig. 3a). Next, the survival rates of these mice were examined in a small population. All *Gad1*^+/+^ mice (total n = 15) survived until 8 weeks of age (Fig. 3b). Two *Gad1*^tTA/+^ mice (total n = 15), one *Gad1*^STOP−tetO/+^ mouse (total n = 17), and eight *Gad1*^tTA/STOP−tetO^ mice (total n = 20) died within 8 weeks after birth (Fig. 3b). The proportion of genotypes in our breeding colony (total n = 1,319) at the weaning period (P21–P28) is shown in Fig. 3c. These observations indicate that the survival rate of *Gad1*^tTA/STOP−tetO^ mice was lower than that of mice with the other genotypes. The protein levels of GAD67 in the FCX (*F*(3,8) = 4.355, *p* = 0.043, one-way ANOVA) and HIP (*F*(3,8) = 10.527, *p* = 0.004, one-way ANOVA) were significantly lower in *Gad1*^tTA/STOP−tetO^ mice than *Gad1*^+/+^ mice at 8 weeks of age (Fig. 3d, e). On the other hand, the protein levels of GAD67 in the CER were not significantly different among the genotypes (*F*(3,8) = 2.867, *p* = 0.104, one-way ANOVA) (Fig. 3f).

**Fig. 4:**
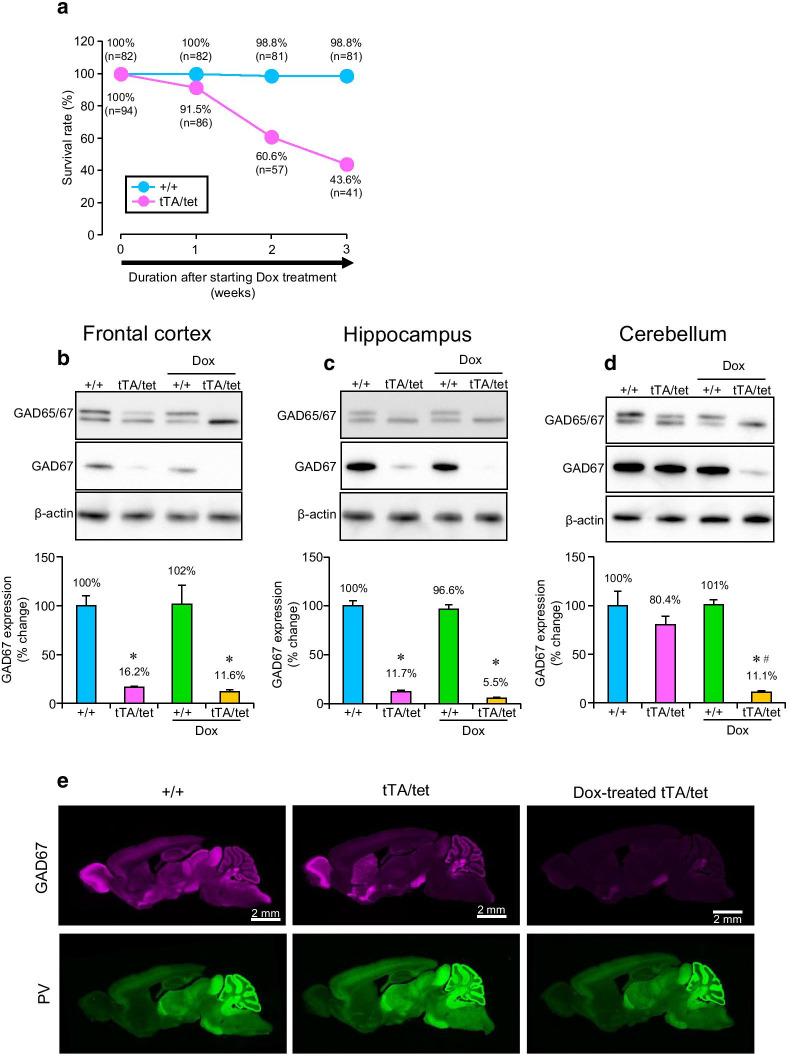
Characterization of *Gad1*^tTA/STOP−tetO^ mice in the presence of Dox treatment. **a** The survival rates of adult *Gad1*^+/+^ (+/+) and *Gad1*^tTA/STOP−tetO^ (tTA/tet) mice after starting Dox treatment. **b**–**d** Representative western blot analysis of the expression of GAD65/67 and β-actin proteins in the frontal cortex (**b**), the hippocampus (**c**) and the cerebellum (**d**) of *Gad1*^+/+^ and *Gad1*^tTA/STOP−tetO^ mice treated and not treated with Dox. β-Actin was used as a loading control. Three independent mice in the respective groups were examined. The band densities of GAD67 and β-actin were analyzed by ImageJ software. GAD67 protein levels were normalized to β-actin protein expression, and the % change was calculated relative to *Gad1*^+/+^ mice. The means with SE are demonstrated as columns. *p < 0.05 vs. the value of nontreated *Gad1*^+/+^ mice (Bonferroni test). ^#^*p* < 0.05 vs. the value of nontreated *Gad1*^tTA/STOP−tetO^ mice (Bonferroni test). **e** Representative immunoreactivities of GAD67 and parvalbumin (PV) proteins in the brain sections of nontreated *Gad1*^+/+^ mice, nontreated *Gad1*^tTA/STOP−tetO^ mice and Dox-treated *Gad1*^tTA/STOP−tetO^ mice. The white bars in the images indicate 2 mm length

Next, the expression levels of GAD67 protein in the brains of *Gad1*^tTA/STOP−tetO^ mice were examined in the absence and presence of Dox treatment. The Dox treatment was performed on the mice at 8–10 weeks of age. We noticed that some *Gad1*^tTA/STOP−tetO^ mice died during the 3 weeks after starting the Dox treatment. Approximately 44% of *Gad1*^tTA/STOP−tetO^ mice survived just after 3 weeks of Dox treatment (Fig. 4a). The expression levels of GAD67 protein in the FCX (*F*(3,8) = 20.563, *p* < 0.001, one-way ANOVA), HIP (*F*(3,8) = 189.298, *p* < 0.001, one-way ANOVA) and CER (*F*(3,8) = 22.760, *p* < 0.001, one-way ANOVA) were significantly decreased by treatment with Dox in *Gad1*^tTA/STOP−tetO^ mice compared with *Gad1*^+/+^ mice (Fig. 4b–d). Importantly, the expression level of GAD67 protein in the CER of *Gad1*^tTA/STOP−tetO^ mice was markedly decreased in the presence of Dox compared with in the absence of Dox (Fig. 4d). By immunofluorescence, GAD67 immunoreactivity in *Gad1*^+/+^ mice was detected widely in the brain, particulary at high levels in the olfactory bulb, globus pallidum, olfactory tubercle, substantia nigra, superior and inferior colliculi, and deep cerebellar nuclei (Fig. 4e, upper panel). In brains of *Gad1*^tTA/STOP−tetO^ mice, the overall immunoreactivity was reduced moderately without Dox treatment and severely with the treatment. Dox treatment to *Gad1*^+/+^ mice did not affect GAD67 immunoreactivity (data not shown). PV is expressed in a major subclass of GAD67-positive inhibitory neurons [32]. No discernible changes in PV immunoreactivity were found between *Gad1*^+/+^ mice and *Gad1*^tTA/STOP−tetO^ mice with or without Dox treatment (Fig. 4e, lower panel). We assessed 3 independent mice in the respective groups and observed similar findings. These results suggest that Dox treatment globally suppresses the expression of GAD67 in the brains of *Gad1*^tTA/STOP−tetO^ mice.

From these experimental results, GAD67 protein levels were found unexpectedly low in several brain regions in *Gad1*^tTA/STOP−tetO^ mice. Therefore, we stopped comparing Dox-treated and Dox-untreated *Gad1*^tTA/STOP−tetO^ mice. Instead, in the following experiments, we compared Dox-treated *Gad1*^tTA/STOP−tetO^ mice and *Gad1*^+/+^ mice.

We measured the brain contents of GABA and glutamate in Dox-treated *Gad1*^tTA/STOP−tetO^ mice and *Gad1*^+/+^ mice. The GABA content was significantly lower in Dox-treated *Gad1*^tTA/STOP−tetO^ mice than in *Gad1*^+/+^ mice in the FCX, HIP and CER (Table 1). On the other hand, the glutamate content in the respective brain regions was comparable between these genotypes (Table 1).

**Table 1:** Brain GABA and glutamate contents

	Contents (nmol/mg tissue)		% Reduction	*t* values	*p* values
	*Gad1*^+/+^	*Gad1*^tTA/STOP−tetO^			
***GABA***					
Frontal cortex	0.632 ± 0.027	0.462 ± 0.019	27.0	5.052	< 0.001
Hippocampus	0.850 ± 0.033	0.546 ± 0.019	35.8	7.844	< 0.001
Cerebellum	0.651 ± 0.026	0.281 ± 0.011	56.8	12.753	< 0.001
***Glutamate***					
Frontal cortex	5.550 ± 0.112	5.617 ± 0.156	− 1.2	0.350	0.731
Hippocampus	5.477 ± 0.148	5.479 ± 0.171	0.0	0.008	0.994
Cerebellum	4.184 ± 0.086	4.356 ± 0.127	− 4.1	1.144	0.268

Dox-treated *Gad1*^+/+^ (n = 10) and *Gad1*^tTA/STOP−tetO^ (n = 9) mice were used to determine the brain contents of GABA and glutamate. Data represent the mean ± SE. % Reduction was calculated by 100 − [(the mean value of *Gad1*^tTA/STOP−tetO^ mice)/(the mean value of *Gad1*^+/+^ mice)] × 100. A *p* value less than 0.05 was considered a significant difference (Student’s *t*-test)

### Behavioral abnormalities in GAD67 knockdown mice

We compared the behavioral phenotypes of *Gad1*^tTA/STOP−tetO^ mice with those of *Gad1*^+/+^ mice in the presence of Dox treatment. To avoid the effects of sex differences, male mice were only used in the following experiments.

**Fig. 5:**
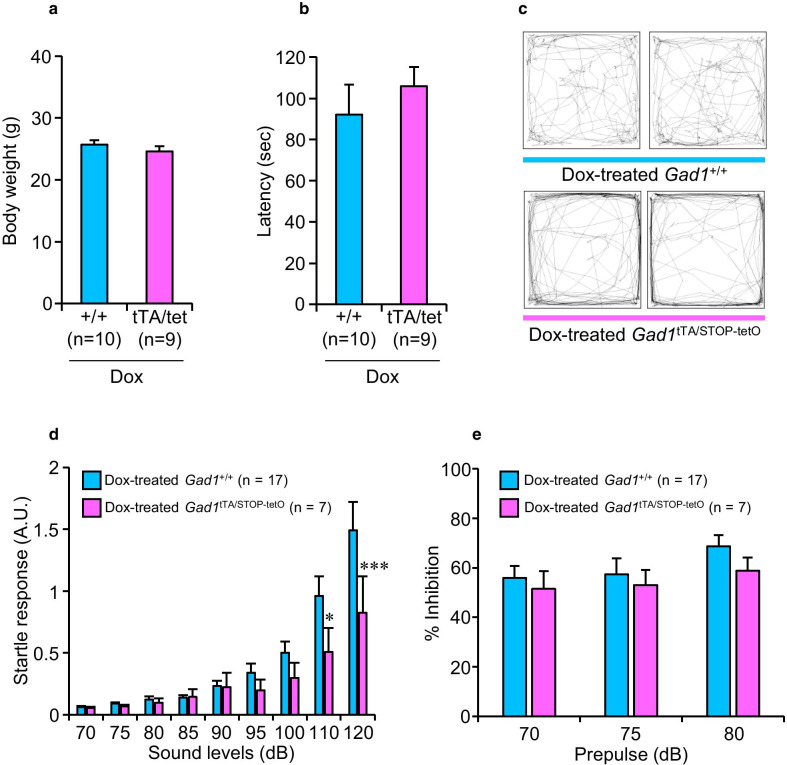
Behavioral consequences of GAD67 knockdown in mice. **a**, **b** Body weights and latency to fall from a cylinder in the rotarod test for Dox-treated *Gad1*^+/+^ and *Gad1*^tTA/STOP−tetO^ mice. **c** Examples of the path traveled in the open-field test by two *Gad1*^+/+^ and two *Gad1*^tTA/STOP−tetO^ mice treated with Dox. **d** Acoustic startle responses of Dox-treated *Gad1*^+/+^ and *Gad1*^tTA/STOP−tetO^ mice. Startle amplitudes by the sounds indicate the startle responses (A.U.). *p < 0.05 and ***p < 0.001 between genotypes (simple main effect of two-way ANOVA). **e** PPI responses of Dox-treated *Gad1*^+/+^ and *Gad1*^tTA/STOP−tetO^ mice. PPI was defined as the percent decline in the startle response (% Inhibition)

We first investigated the body weights and the performance of motor coordination in Dox-treated *Gad1*^tTA/STOP−tetO^ and *Gad1*^+/+^ mice. No difference was observed in the body weights (*t*(17) = 1.066, *p* = 0.301, Student’s *t*-test, Fig. 5a) or the latency to fall from the cylinder in the rotarod test (*t*(17) = 0.772, *p* = 0.451, Student’s *t*-test, Fig. 5b) between the two genotypes.

We next conducted the open-field test, which is a well-accepted behavioral test to evaluate the anxiety-like state of rodents [33]. The total distance, total duration of movement, moving speed, distance per movement, and duration per movement were significantly increased in Dox-treated *Gad1*^tTA/STOP−tetO^ mice compared with Dox-treated *Gad1*^+/+^ mice (Table 2). In contrast, the total number of movement episodes was significantly decreased in Dox-treated *Gad1*^tTA/STOP−tetO^ mice compared with Dox-treated *Gad1*^+/+^ mice (Table 2). These observations indicate that *Gad1*^tTA/STOP−tetO^ mice walk longer distances with less frequency. In addition, Dox-treated *Gad1*^tTA/STOP−tetO^ mice walked a long time in the wall side and a short time in the center region compared with Dox-treated *Gad1*^+/+^ mice (Table 2 and Fig. 5c), indicating that *Gad1*^tTA/STOP−tetO^ mice exhibited anxiety-like behavior in the open-field test.

**Table 2:** Summary of exploratory behaviors in the open-field test

	*Gad1*^+/+^	*Gad1*^tTA/STOP−tetO^	*t* values	*p* values
Total distance (cm)	2434.4 ± 102.7	3495.0 ± 179.7	5.389	< 0.001
Total duration of movement (s)	166.8 ± 4.9	185.7 ± 5.5	2.235	0.036
Total number of movement episode	95.4 ± 2.1	86.7 ± 2.6	2.330	0.029
Moving speed (cm/s)	13.1 ± 0.3	17.8 ± 0.7	6.784	< 0.001
Distance per movement (cm)	23.4 ± 1.3	38.7 ± 3.1	5.380	< 0.001
Duration per movement (s)	1.8 ± 0.1	2.2 ± 0.1	3.295	0.003
Time spent in the wall side (s)	246.9 ± 5.5	271.6 ± 5.3	2.671	0.014
Time spent in the center region (s)	53.1 ± 5.5	28.4 ± 5.3	2.671	0.014
% Time spent in the center region	17.7 ± 1.8	9.5 ± 1.8	2.670	0.014

Exploratory behaviors were measured for 5 min in the open-field in Dox-treated *Gad1*^+/+^ (n = 17) and *Gad1*^tTA/STOP−tetO^ (n = 7) mice. Data represent the mean ± SE. A *p* value less than 0.05 was considered a significant difference (Student’s *t*-test)

We further assessed acoustic startle responses and PPI responses in Dox-treated *Gad1*^tTA/STOP−tetO^ and *Gad1*^+/+^ mice. The PPI response provides an operational index of sensorimotor gating, and an impaired PPI response is observed in subjects with schizophrenia [34]. The amplitude of acoustic startle responses was significantly affected by the effect of genotype × sound level interaction (*F*(8,168) = 2.745, *p* = 0.007, two-way ANOVA). The simple main effect of genotypes was statistically significant at the sound levels of 110 dB (*F*(1, 92) = 6.345, *p* = 0.014) and 120 dB (*F*(1, 92) = 13.550, *p* < 0.001) (Fig. 5d). The PPI responses were significantly affected by the effect of prepulse intensity (*F*(2,42) = 6.713, *p* = 0.003, two-way ANOVA) but not by the effect of genotype × prepulse intensity interaction (*F*(2,42) = 0.577, *p* = 0.566, two-way ANOVA) or genotype (*F*(1,21) = 0.514, *p* = 0.481, two-way ANOVA) (Fig. 5e).

## Discussion

We first generated *Gad1*^STOP−tetO^ knock-in mice. All homozygous *Gad1*^STOP−tetO/STOP−tetO^ mice died on the day of birth, and 57% of *Gad1*^STOP−tetO/STOP−tetO^ mice exhibited a cleft palate. The expression of GAD67 protein was lacking in the brains of *Gad1*^STOP−tetO/STOP−tetO^ mice with or without the cleft palate. These phenotypes in *Gad1*^STOP−tetO/STOP−tetO^ mice are consistent with those in *Gad1*^−/−^ mice [15, 31]. Therefore, the function of the *Gad1* gene was eliminated by the insertion of the Neo-STOP-tetO cassette in the 5′-untranslated region of the *Gad1* gene in mice. It has been reported that neonatal death in *Gad1*^−/−^ mice is caused by respiratory failure rather than impairment of suckling [15, 35]. Therefore, neonatal death in *Gad1*^STOP−tetO/STOP−tetO^ mice may also be caused by respiratory failure.

We next developed *Gad1*^tTA/STOP−tetO^ biallelic knock-in mice by crossing *Gad1*^tTA/+^ and *Gad1*^STOP−tetO/+^ parents. Approximately 40% of *Gad1*^tTA/STOP−tetO^ mice died on the day of birth, and the number of *Gad1*^tTA/STOP−tetO^ mice at P21-P28 in our breeding colony was smaller than the numbers of mice with the other genotypes. None of the *Gad1*^tTA/STOP−tetO^ mice demonstrated a cleft palate. Unexpectedly, some adult *Gad1*^tTA/STOP−tetO^ mice died by treatment with Dox. Therefore, GAD67 is important for survival not only in the neonatal period but also in adulthood. However, the cause of death in Dox-treated *Gad1*^tTA/STOP−tetO^ mice is currently unknown. To resolve this question, pathological examination is required in a future study.

Adult mice with *Gad1* haplodeficiency demonstrated an approximately 40% reduction in GAD67 protein levels in the whole brain compared with *Gad1*^+/+^ mice [27]. Consistently, we observed that heterozygous *Gad1*^tTA/+^ and heterozygous *Gad1*^STOP−tetO/+^ knock-in mice exhibited a 30–50% reduction in GAD67 protein levels in the FCX, HIP and CER compared with *Gad1*^+/+^ mice. In the absence of Dox treatment, the expression level of GAD67 protein in *Gad1*^tTA/STOP−tetO^ mice relative to *Gad1*^+/+^ mice was dependent on the brain regions. In the immunoblotting analysis, the expression of GAD67 protein in the CER was comparable between *Gad1*^tTA/STOP−tetO^ mice and *Gad1*^+/+^ mice. However, the expression of GAD67 protein in the FCX and HIP was significantly lower in *Gad1*^tTA/STOP−tetO^ mice than *Gad1*^+/+^ mice. Importantly, in the presence of Dox treatment, GAD67 expression was reduced by approximately 90% in the brains of *Gad1*^tTA/STOP−tetO^ mice, compared with Dox-treated *Gad1*^+/+^ mice. The brain-wide reduction of GAD67 expression in Dox-treated *Gad1*^tTA/STOP−tetO^ mice was also observed in the immunofluorescence analysis. These findings suggest that GAD67 expression is suppressed by treatment with Dox in the brains of *Gad1*^tTA/STOP−tetO^ mice.

In adult mice with *Gad1* haplodeficiency, the GABA content in the brain was reduced by 7–20% from those in wild-type control mice [15, 32]. In this study, we found that the GABA contents in the FCX, HIP and CER of Dox-treated *Gad1*^tTA/STOP−tetO^ mice were reduced by 27.0–56.8% from those in Dox-treated *Gad1*^+/+^ mice. This result is inferred that the amount corresponding to the reduced GABA content compared to the GABA content in the brain of *Gad1*^+/+^ mice is derived from the amount biosynthesized by GAD67 expression in the brain of *Gad1*^tTA/STOP−tetO^ mice. Therefore, the GABA reduction in the brains of Dox-treated *Gad1*^tTA/STOP−tetO^ mice was larger than that in *Gad1* haplodeficient mice. Because approximately half of the brain GABA is produced by GAD65 in adulthood [36], the remaining GABA in the brain of Dox-treated *Gad1*^tTA/STOP−tetO^ mice is mainly synthesized by GAD65. The brain glutamate contents in Dox-treated *Gad1*^tTA/STOP−tetO^ mice were comparable to those in Dox-treated *Gad1*^+/+^ mice. Therefore, the glutamatergic system may be normal in Dox-treated *Gad1*^tTA/STOP−tetO^ mice.

GAD67 haplodeficient mice demonstrated several abnormal behaviors, such as hyper-locomotor activity, reduced interactions with an unfamiliar mouse, and aggressive behavior. However, the emotional behaviors in *Gad1* haplodeficient mice were normal in the open-field test, the light–dark avoidance test and the elevated plus-maze test [21, 23]. In the current open-field test, Dox-treated *Gad1*^tTA/STOP−tetO^ mice walked longer distances than Dox-treated *Gad1*^+/+^ mice. In addition, Dox-treated *Gad1*^tTA/STOP−tetO^ mice preferentially walked for more time along the walls and for less time in the center region. These observations indicate that Dox-treated *Gad1*^tTA/STOP−tetO^ mice exhibited behavioral abnormalities, including the hyper-locomotor activity and anxiety-like behavior, in the open-field test. Since *Gad1*^tTA/STOP−tetO^ mice exhibited normal body weight and motor coordination in the presence of Dox treatment, the changes in exploratory behavior are unlikely to be associated with physical dysfunction. It is well accepted that inhibition of GABAergic tone elicits anxiety-like behavior in the open-field test [33]. Therefore, the reduction in brain GABA in Dox-treated *Gad1*^tTA/STOP−tetO^ mice may cause the induction of anxiety-like behavior. The mice lacking GAD67 in protein phosphatase 1 regulatory subunit 2 (Ppp1r2)-expressing cells, in which Cre recombinase expression is largely confined to GABA interneurons of the cerebral cortex and the hippocampus, demonstrated the hyper-locomotor activity and anxiety-like behavior in the open-field test [37]. In addition, we recently reported that mice lacking GAD67 in somatostatin-expressing GABA interneurons demonstrated anxiety-like behavior in the open-field test without affecting locomotor activity [20]. Therefore, the anxiety-like behavior in Dox-treated *Gad1*^tTA/STOP−tetO^ mice may be due to GAD67 knockdown from somatostatin-expressing GABA interneurons in cortical and hippocampal areas. In addition, the hyper-locomotor activity in Dox-treated *Gad1*^tTA/STOP−tetO^ mice may be associated with GAD67 knockdown from the other subtypes of GABA interneurons.

In this study, Dox-treated *Gad1*^tTA/STOP−tetO^ mice showed a normal response to PPI. Therefore, global knockdown of GAD67 does not affect the PPI response. However, we previously reported that mice with conditional *Gad1* heterozygous knockout predominantly in parvalbumin-positive cells demonstrated an impaired response to PPI [19]. It is possible that a mild reduction in GAD67 in parvalbumin-positive cells might be required for impairing the PPI response. Interestingly, the startle responses elicited by large acoustic stimuli were impaired in Dox-treated *Gad1*^tTA/STOP−tetO^ mice. The acoustic startle response is a simple reflex of animals that results in a whole body motor response elicited by hearing a loud sound. Multiple neural circuits from the cochlear nucleus to motoneurons contributes to elicit the acoustic startle reflex [38]. Therefore, it is possible that Dox-treated *Gad1*^tTA/STOP−tetO^ mice might exhibit the impairment of the acoustic startle reflex associated with those neural circuits. In addition, GABAergic interneurons expressing GAD67 proteins constitute auditory neural networks and contribute to auditory function [39–41]. Therefore, global knockdown of GAD67 in the brain might induce the impairment of auditory function. To note that the behavioral responses by intensity of pre-pulse in the control mice were different between the present study and the previous study [19]. There are several possible reasons to explain the different observations. First, it may be related to the difference in the environment during development between these two types of mice because all mice in the present study were born from GAD67 haplodeficient (*Gad1*^STOP−tetO/+^) mothers and cared by the same mothers. We previously reported that wild-type (*Gad1*^+/+^) mice born from GAD67 haplodeficient (*Gad1*^GFP/+^) mothers exhibited the vulnerability to stress at the adult age [42]. In addition, it has been reported that the anxious state in mother mice affects behavioral phenotypes in their pups at the adult age [43]. Second, we used wild-type (*Gad1*^+/+^) mice as the control, but Fujihara et al. [19] used *Gad1*^flox/+^ mice as the control. The difference in genotypes might affect the reaction of PPI in mice. Third, there is difference whether the mice were subjected to Dox treatment or not. It can not be excluded the possibility that the Dox treatment affected the PPI reaction in mice. Further experiments are necessary to resolve the difference in the baseline reaction of pre-pulse between the studies.

The *GAD1* gene and GAD67 protein have often been targeted in human studies to elucidate an association with the pathophysiology of psychiatric disorders [44–46]. Reduced full-length *GAD1* transcript and GAD67 protein is a consistent finding in the postmortem brains of patients with several psychiatric disorders including schizophrenia, bipolar disorder and major depressive disorder [7–12]. Because GAD67 reduction was predominantly observed in parvalbumin-positive GABAergic interneurons in the postmortem brains of schizophrenic patients [47–49], mice with conditional knockout of GAD67 in parvalbumin-positive cells have been used as an animal model of schizophrenia [18, 19, 50, 51]. On the other hand, the specific subsets of GABAergic interneurons reducing GAD67 expression have not yet been identified in other psychiatric disorders, which might indicate that GAD67 expression is globally reduced in the brain of subjects with the other psychiatric disorders. In this study, global knockdown of GAD67 elicited anxiety-like behavior in mice. Therefore, we suggest the possibility that the global reduction in *GAD1* transcript and GAD67 protein in the brain might be related to the occurrence of anxiety symptoms frequently comorbid in several psychiatric disorders.

We did not examine whether administration of anxiolytic, antidepressant or antipsychotic drugs improves anxiety-like behavior in Dox-treated *Gad1*^tTA/STOP−tetO^ mice. Because of the high lethality rate, we could not immediately prepare a sufficient number of animals for conducting a pharmacological study. Therefore, we will determine the utility of Dox-treated *Gad1*^tTA/STOP−tetO^ mice as a tool for screening potential medications for anxiety symptoms in a future study.

In summary, *Gad1*^tTA/STOP−tetO^ biallelic knock-in mice showed GAD67-knockdown phenotypes when treated with Dox. We suggest that the global reduction in GAD67 elicits emotional and auditory abnormalities in mice. The use of GAD67 knockdown mice will provide new insights into the neurobiological impact of GAD67 dysfunction and elucidate the neurobiological mechanisms of emotional abnormalities associated with psychiatric disorders.

## Data Availability

All data are available within the manuscript.

## Abbreviations

GAD67: Glutamate decarboxylase 67
GAD65: Glutamate decarboxylase 65
Dox: Doxycycline
GABA: γ-Aminobutyric acid
tTA: Tetracycline-controlled transcriptional activator
tetO: Tetracycline operator site
FCX: Frontal cortex
HIP: Hippocampus
CER: Cerebellum
CP: Cleft palate
PV: Parvalbumin
PPI: Prepulse inhibition
ANOVA: Analysis of variance
SE: Standard error
